# The Profile of Belgian Osteopaths: A Cross-Sectional Survey

**DOI:** 10.3390/healthcare10112136

**Published:** 2022-10-27

**Authors:** Patrick L.S. van Dun, Johan Verbeeck, Lorenzo Arcuri, Jorge E. Esteves, Francesco Cerritelli

**Affiliations:** 1 Clinical-Based Human Research Department, Foundation COME Collaboration, 65121 Pescara, Italy; 2 Belgium National Centre, Foundation COME Collaboration, 2800 Mechelen, Belgium; 3 Independent Statistical Consultant, 2830 Blaasveld, Belgium

**Keywords:** osteopathy, osteopathic medicine, workforce survey

## Abstract

Background: This study gives an update on the characteristics of Belgian osteopaths five years after the Benelux Osteosurvey. Additional new data were collected on their professional identity and views on the profession. Methods: All Belgian osteopaths who could be contacted (*n* = 1473) were invited to complete a voluntary, online-based, closed-ended survey distributed between May and September 2018. The survey, composed of 52 questions and seven sections, was formally translated from English to Dutch and French and adapted from the original version. Adult, self-defined osteopaths working in Belgium were eligible. Recruitment of participants was performed through all professional associations and the InterMutualistic Agency. Descriptive statistics were used to analyse the data. Results: The survey was completed by 332 osteopaths. Thirty-one per cent of the respondents were female. Almost all the respondents were self-employed (99.4%); half of them worked as part of a team (47.6%). Most respondents had a 5-year part-time training, and the majority had a previous academic degree, mainly in physical therapy (65.8%). According to respondents, most patients seek care for lumbar non-specific low back, pelvis and neck pain. Most respondents strongly define themselves as osteopaths and advertise themselves exclusively as osteopaths. Conclusions: This survey provided an update of the current characteristics of Belgian osteopathic practitioners and added new information on their professional identity and views on the profession. The information provided could contribute to the body of evidence used by stakeholders and policymakers in the future regulation of the profession in Belgium.

## 1. Introduction

Osteopathy has been practised in Belgium for more than 50 years [1]. Although nonconventional medical practices, such as osteopathy, have been governed by law in Belgium since 1999 [2], this law has not yet been implemented. Osteopathy therefore is officially recognised in Belgium, but still not yet regulated. Of all non-conventional medical practices considered in the Colla law (acupuncture, chiropractic, homoeopathy and osteopathy), only osteopathy increased the number of Belgians consulting from 3.9% in 2001 to 8.4% in 2018, making it by far the most consulted non-conventional medical practice [3].

To better understand osteopathy and chiropractic in Belgium, the Ministry of Health commissioned a survey in 2010 [4]. The Belgian Healthcare Knowledge Centre (KCE) survey was sent to all osteopaths, members of professional associations in Belgium at the time, representing nearly 70 per cent of the country’s total osteopathy population. According to the findings of this survey, osteopathy appeared to be a more diverse non-conventional medical practice than chiropractic, both for techniques and approach. Only three years later, an osteopathic joint research organisation decided to conduct a new survey, because the KCE survey focused on both chiropractors and osteopaths and on mainly technical aspects of the osteopathic profession. However, there was the need for the osteopathic profession to obtain more information regarding, safety aspects, diagnosis and therapy of internal or sensitive areas and the use of additional diagnostic and therapeutic techniques. In addition, the osteopathic profession wanted to open the survey to anyone who presented themselves as an osteopath and not only to members of an osteopathic professional association. Therefore, in 2013, a Belgian and a Dutch research organisation (Commission for Osteopathic Research, Practice and Promotion and Stichting Wetenschappelijk Osteopathisch Onderzoek), with the support of all osteopathic professional organisations in the Benelux (Belgium, the Netherlands and Luxembourg), surveyed all Benelux osteopaths concluding that, despite some regional similarities, they showed unique characteristics [5].

In the last few years, similar surveys have been performed in other European countries, such as Spain, Switzerland, Italy, Germany, the UK, Portugal and Austria, describing osteopathic practitioners and the care they provide [6,7,8,9,10,11,12,13].

While the KCE survey data were used to inform the Ministry of Health about the state of the manual professions of osteopathy and chiropractic, the Benelux Osteosurvey provided valuable information for the profession of osteopathy to further professionalise and advocate regulation to the government. This information now needs to be updated to characterise the profile of Belgian osteopaths. The Osteopathic Practitioners, Estimates and RAtes (OPERA) project of the Centre of Osteopathic MEdicine Collaboration (COME), was chosen for this purpose. OPERA, as an international survey project, was already carried out in Austria, Italy, Portugal and Spain [9,11,12,13,14] and lends itself well to profiling the osteopathic profession and enabling international comparison. The questionnaire used in OPERA was based on that of the Benelux Osteosurvey [5] with omissions of some original questions that did not seem to contribute essential information and the omission/adaptation of others with questioned validity. In addition, a whole new section with information about identity and perceptions of the profession, which were not covered in previous surveys in Belgium was added [4,5]. The scientific purpose is to estimate the current size and dimension of osteopathy in Belgium to characterise the profession and define its role in national public health systems. This will enable informed decision-making for policies and strategies by professional associations, statutorily regulatory authorities, governmental departments and academic institutions to favour a reflective professional approach and use nationally collected data as benchmarks to set comprehensive and achievable goals for improvement.

## 2. Methods

This cross-sectional survey was conceived as a quantitative descriptive research design using a practitioner-based anonymous online survey, distributed throughout Belgium between May and September 2018. The methodology used was reported in detail in prior OPERA studies [9,11,12,13,14]. The reporting guideline utilised was the SUrvey Reporting GuidelinE (SURGE) [15].

The intended sample consisted of Belgian osteopaths who were fluent in Dutch or French, irrespective of their training and academic degrees. The study excluded osteopathic students, including those enrolled in higher education undergraduate programmes.

A separate website was created for this study with general information, FAQs and a hyperlink for participation [16]. All four officially recognised osteopathic professional associations were contacted and informed about the survey and asked for their support to recruit their members. In this way, 1218 osteopaths were personally contacted to participate in the survey. An e-campaign was set up with information that could be freely used by the professional associations via social media, the internet and newsletters to encourage their members to participate in the study.

The InterMutualistic Agency (an agency that groups seven Belgian health insurance funds) was contacted in order to reach osteopaths who were not members of a professional association. In this way, 255 more osteopaths could be contacted and informed about the survey.

During the six-month recruitment period, e-flyers were consistently sent to different mailing lists to encourage participation in the survey. It consisted of notifications posted in the channels of professional associations on social media. These posts contained a link to a page on the OPERA website with details about the survey. If the person accepted the conditions outlined to participate in the research, they could then enter their email address. The IT system would mail the questionnaire link to the specified email address. The server-based information technology system (COME Survey) permits the monitoring of potential duplicate participation. Upon registration, each participant was requested to enter a reliable, verifiable email address. Respondents who successfully registered received an email containing a unique web link to the survey, allowing them to participate. A subsequent attempt with the exact email account would be denied. The entire mailing list was sent a reminder to disregard the email if they had already responded. Participation in the research was entirely voluntary.

The OPERA survey was based on that of the Benelux Osteosurvey [5] but was modified based on additional elements described in the OPERA research conducted in Spain [12]. The survey consisted of a total of 52 questions divided into seven parts: socio-demographics, job characteristics and professional activities, training and continuous learning, professional identity, costs and features of clinical practice with respect to consultation structure, patient profile and osteopathic skills. All questions were structured as close-ended questions with options provided. Five questions were mixed (i.e., they contained an open-ended option as well). Data from these open-ended options were not considered for further analysis but were an additional consideration for future surveys. Fifteen questions utilised a five-point Likert-scale.

The translation of the original English version into French and Dutch fulfilled the “forward-backward” process encouraged by the World Health Organisation and described in other OPERA studies [9,11,12,13]. A pilot study was conducted with 16 osteopaths, representing the various professional associations, to validate the questionnaire. In response to feedback received from this group, modifications in language used were made.

Previously developed, the OPERA survey online platform utilised a data warehouse for research purposes. Following directive 2018/1725CE of the European Parliament, the questionnaire fulfilled the data privacy and anonymity. Therefore, the data was anonymised and transmitted using the COME Survey software [9]. The latter conducts research dealing with sensitive data in a highly secure manner [9]. Answers were rendered anonymous, and IP addresses were not either revealed or made accessible. The system manages the link among email addresses, research ID, as well as survey status automatically so that no one can identify the responses provided. Only the OPERA study group had access to comprehensive and anonymous data. The data is kept for five years and utilised for additional studies including benchmarking.

Participants were emailed information about the study, and after registering, they obtained informed consent as well as a questionnaire link to fill out the survey by providing data from the seven sections mentioned above.

Answers of completed questionnaires were collected while avoiding easy identification. This includes an age range, limited information regarding training institutions and a large geographical working area. In addition, no sensitive information was collected from respondents, including their names/surnames, birth date, fiscal details, residence or workplace addresses, as well as ethnic, biometric, genetic, racial, healthcare and gender orientation information.

The R statistical programme (version 3.1.3) was utilised for descriptive analysis using frequencies and percentages and two-sided tests (=0.05).

On the survey access page, respondents were required to consent to their participation. The Institutional Review Board of the Foundation COME Collaboration approved the survey (January 2018). 

## 3. Results

A total of 332 of the 1473 contacted osteopaths completed the survey (response rate: 22.5%). The age, gender and province location of the respondents are representative for the Belgian osteopaths when compared to the data of the professional associations. Osteopaths who are not affiliated with a professional association are however underrepresented among the respondents (2.4% responders versus 17.3% of the contacted osteopaths), while members of Osteopathie.be are overrepresented (74.9% responders versus 55.5% of the contacted osteopaths). The response rate of members of a professional association is clearly higher compared to the response rate of non-members (26.6% versus 3.1%).

### 3.1. Socio-Demographic Characteristics

**Figure 1:**
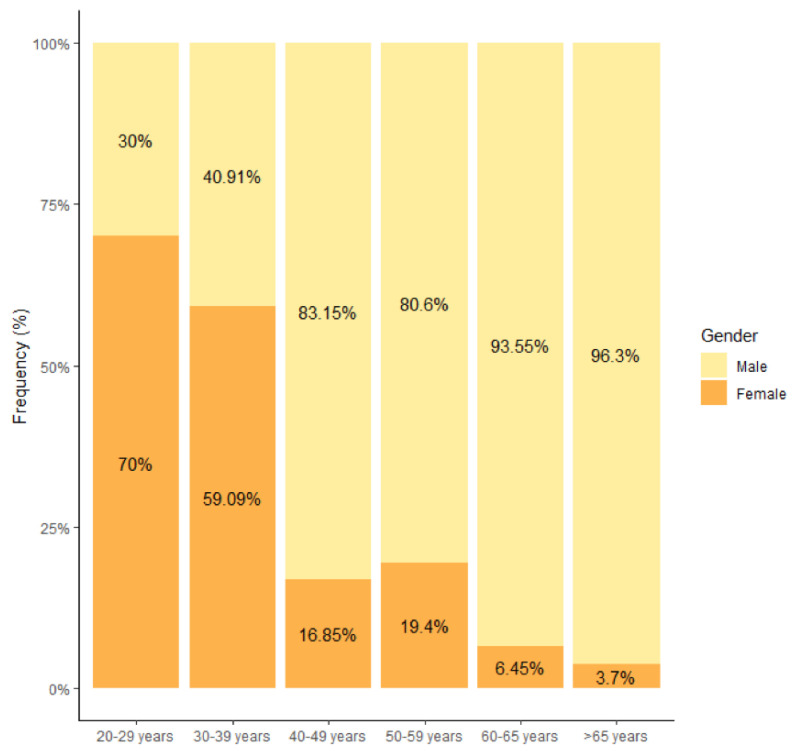
Age distribution by gender (*n* = 332).

Among the 332 responders, 228 were male (68.7%). Thirty-nine per cent of the Dutch-speaking respondents were female, compared to 22.6% of the French-speaking respondents (*p* = 0.002). A marked gender shift was observed in the 30–39 years category, showing more women in the lower age categories (Figure 1). The gender shift in this category is entirely due to the Dutch-speaking respondents. French-speaking respondents only showed a gender shift in the 20–29 age category. The majority of survey participants were between the ages of 30 and 49 (53.3%). For each participant over the age of 65, there were 1.10 people between the ages of 20 and 29. Table 1 displays the distribution by age, gender and culture.

Most respondents had a Belgian nationality (91.0%), followed by French (6.3%) and Dutch (2.1%). Overall, all Belgian provinces were represented in this survey’s responses., following the provincial dispersion in the professional associations, with most respondents from Antwerpen (16.6%), followed by West-Vlaanderen (13.3%), Brussel (13.0%) and Oost-Vlaanderen (12.1%). Almost all respondents (97.6%) were members of a professional osteopathic association (Table S1).

### 3.2. Work Situation and Professional Pursuits

Nearly every participant was self-employed (99.4%), and 81.5% were the sole owners of their clinic (Table 2). Respondents who worked in teams (47.6%) did so mostly with osteopaths (23.7%), physiotherapists (21.2%) and psychologists (13.2%) (Figure S1). The majority of the Dutch-speaking respondents worked as part of a team (52%), whereas 42.6% of the French-speaking respondents did so (*p* = 0.0065). The greatest difference between the two language communities was found in the group who only worked as part of a team (31.6% Dutch-speaking as against 8.4% French-speaking respondents) (*p* ≤ 0.0001). The most preferred team member among the Dutch-speaking respondents is the osteopath (34.4%), while they come second (with 15.8%) to physiotherapists (21.3%) among the French-speaking respondents.

In addition to their clinical osteopathic practice, more than a quarter of respondents (28.6%) disclosed other professional activities. Of them, 25.5% also worked as lecturers in osteopathic undergraduate and postgraduate courses, and 20.8% worked clinically as physiotherapists (Table S2). French-speaking respondents had slightly more other professional activities (32.9% against 24.9%) (*p* = 0.1346) and worked more clinically as physiotherapist (26.7% against 14.9%) (*p* = 0.0573).

**Figure 2:**
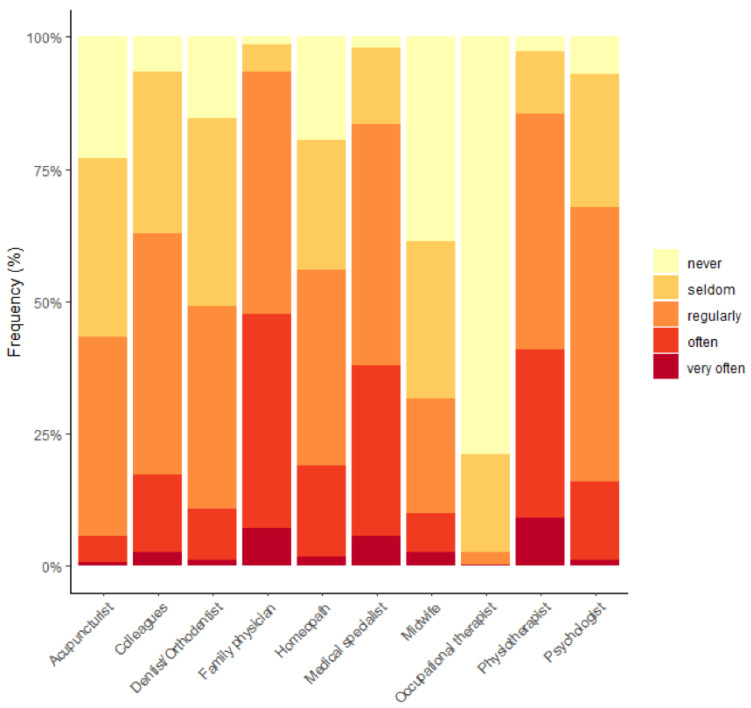
Frequency of referral of patients by osteopaths to other health professionals (%).

**Figure 3:**
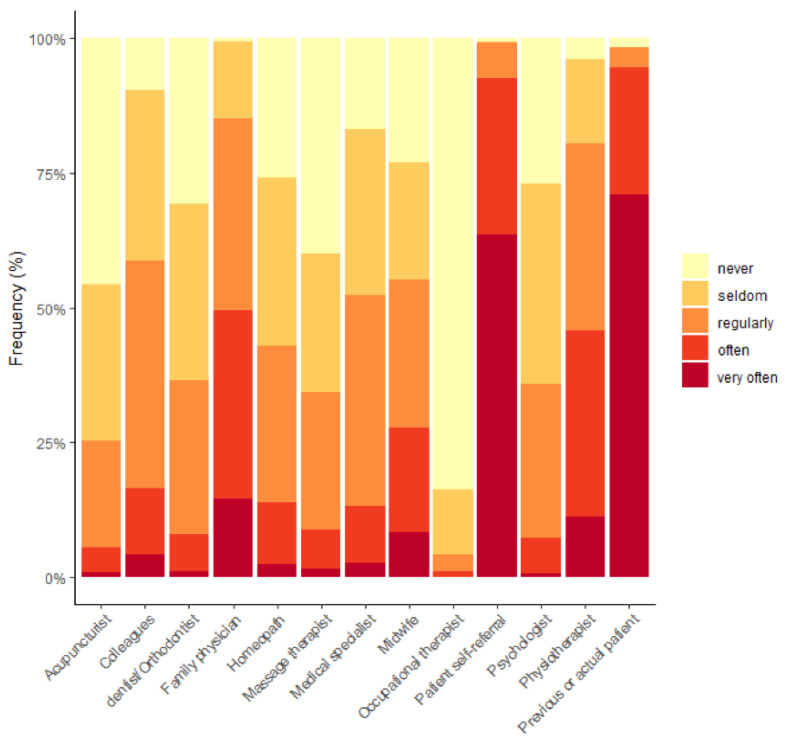
Frequency of referral of patients by other health professionals to osteopaths (%).

Figure 2 shows the frequency of patient referrals by respondents to other health professionals. Respondents also reported on the frequency of referrals received in Figure 3.

Thirty per cent of respondents advertised themselves as treating specific patient populations, such as children (22.6%), newborns (21.0%) and pregnant women (17.4%). The majority of participants communicated with patients about the policy regarding cancellation/missed appointments (68.1%) and confidentiality policy (60.5%). Information about chaperone policy for the examination and treatment of intimate areas, the examination and treatment of minors and data handling policy were mentioned only by 41.3%, 41.3% and 42.2%, respectively.

### 3.3. Osteopathic Training and Continuing Professional Development (CPD)

The predominant form of education in osteopathy was a 5-year (44.9%) part-time training (65.1%) in Belgium (72.0%), concluding with a Diploma in Osteopathy (DO) (65.7%). There is a clear difference between the two language communities: 76.8% of the French-speaking respondents had followed a part-time training, compared to 54.8% (*p* ≤ 0.0001) of the Dutch-speaking respondents. Half of the respondents (51.2%) held a health care master’s degree, of which 16.9% in osteopathy (13.6% Dutch-speaking and 20.7% French-speaking) (*p* = 0.1157) and 34.3% in another health profession (29.9% Dutch-speaking and 39.4% French-speaking) (*p* = 0.0918). The training type shifts in the age category of 30–39 with a full-time program in the younger age categories. For the French-speaking respondents, this was only observed in the 20–29 age category. There is a clear gender difference concerning the type of training, with 62.5% of female respondents having followed a full-time training programme compared to 22.4% of male respondents (*p* ≤ 0.0001). This gender difference is even more pronounced among the Dutch-speaking respondents, with 71.0% of female and 28.7% of male respondents that have followed a full-time training compared to 45.7% (*p* ≤ 0.0001) of female and 16.7% (*p* = 0.0008) of male French-speaking respondents. The overall mean time since graduation was 13 years. The majority of respondents (70.3%) had a preliminary healthcare training, mainly as physiotherapists (65.8%; 60.4% Dutch-speaking and 71.9% French-speaking) (*p* = 0.0075). Surprisingly, 28.0% of the respondents indicated that working in an osteopathic clinical setting was not part of their osteopathic training (20.3% Dutch-speaking and 36.8% French-speaking) (*p* = 0.0014). The majority of respondents (88.9%) had attended CPD courses in the preceding year (Table S3). However, only 27.8% achieved the required annual 16 credit points.

### 3.4. Professional Identity and Views as an Osteopath

Most respondents exclusively advertised themselves as osteopaths (81.6%). Almost all respondents agreed or strongly agreed with the statements on professional identity and thus define themselves as osteopaths, are proud of it and consider it important to be an osteopath (Table S4). Respondents agreed and strongly agreed that osteopathy should be regulated as primary medical practice (88.8%) and considered that osteopathy should be regulated by law as an independent profession (89.2%), whereas 73.2% of them think that regulation would have a positive impact on how osteopathic professionals practise. Only 25.9% of respondents strongly agreed that the quality of patient care provided by osteopaths in Belgium is generally good, while 62% agreed. Most respondents agreed and strongly agreed that patients should get better remuneration for osteopathic care (88%) and that there should be better collaboration with other health professionals (89.5%) (Table S5).

### 3.5. Fees and Consultation Features

The majority of respondents worked five days per week, scheduled 30–45 min for a consultation, and the time to the first appointment was between two to seven working days. The median number of visits per week was 31–35 of which 6 to 10 were new patients. The proportion of respondents who apply different fees for the initial and subsequent consultations is 0.11, with a median fee of EUR 51–60 for the initial consultation and EUR 41–50 for the subsequent consultation. Unlike Dutch-speaking respondents, French-speaking charged a median fee of EUR 51–60 for a subsequent consultation. The median patient consultation rate per week was 26–30 for French-speaking respondents and 41–45 for Dutch-speaking respondents. More detailed information on the key consultation features is shown in Table 3.

### 3.6. Patients

While 64.5% of participants reported that equal numbers of males and females visit their practice, 27.5% stated that more females consulted them. According to survey participants, all age categories were represented among their patients, although they indicated that most were adults, 40–65 years old, consulting most often (65.7% reported ‘very often’), followed by 18–40 (61.5%). While 16.0% of participants never treated infants under the age of two (10.2% of Dutch-speaking and 22.6% of French-speaking respondents) (*p* = 0.0034). In the past year, respondents declared that the main reason for consultation was ‘very often’ for acute (46.7%), followed by chronic (31.3%) complaints. Respondents estimated the lower spine (81.3%) as the site of primary or main symptoms of their patients, followed by the pelvis (70.5%), neck (69.6%) and upper spine (58.4%). Table 4 reports the most common specific clinical conditions estimated by respondents.

### 3.7. Diagnosis and Treatment

At each consultation, half of the respondents (53.6%) always performed a new clinical examination (46.3% of Dutch-speaking and 61.9% of French-speaking respondents) (*p* = 0.0062), and another 34.3% confirmed they did so often. The majority of respondents (63.9%) confirmed always performing exclusion diagnostics to decide whether or not to treat (50.3% of Dutch-speaking and 79.4% of French-speaking respondents) (*p* ≤ 0.0001). Furthermore, 68.1% of respondents stated that they explained the treatment plan to their patients, but only 56.3% informed them about possible risks and adverse events of the treatment they recommended. Table 5 and Table 6 detail the most common diagnostic and therapeutic procedures. The most striking differences in diagnostic techniques were the greater use of neurological (*p* = 0.0002) and orthopaedic (*p* ≤ 0.0001) tests by the French-speaking respondents (regional difference of 19.5% and 26.2% of the ‘often’ and ‘very often’ scores, respectively) and the greater use of visual inspection (*p* ≤ 0.0001) by the Dutch-speaking respondents (regional difference of 23.1% of the ‘often’ and ‘very often’ scores). The main differences in therapeutic techniques were the greater use of functional (*p* ≤ 0.0001) and Progressive Inhibition of Neuromuscular Structures (PINS) techniques (*p* = 0.0012) by the French-speaking respondents (regional difference of 23.5% and 17.9% of the ‘often’ and ‘very often’ scores, respectively).

Regarding diagnostic and therapeutic procedures for internal and sensitive areas, respondents found intraoral techniques to be the most commonly utilised (38.3% ‘often’ to ‘very often’). Genital and rectal techniques were ‘never’ performed by 63.6% and 48.5% of respondents, respectively. Advice on exercises and stress management were always discussed with patients as part of the treatment plan’s recommendations in 43.7% and 40.1%, respectively. Respondents consider as very important reasons for referring patients to other health professionals when dealing with clinical situations such as: ‘it is not my field of expertise’ (71.1%), ‘indication of an undiagnosed pathology’ (59.3%) and ‘increased level of primary symptoms’ (56.6%). The majority of the respondents (57.2%) do not use supplementary methods in their osteopathic practice. Among those that do, ‘exercise therapy’ (41.6%) and ‘nutrition therapy’ (35.9%) were the most common treatment approaches, while ‘applied kinesiology’ (26.1%) was the most common diagnostic method.

## 4. Discussion

The aim of this survey was to give an update of the professional profile of osteopaths working in Belgium. In general, the typical osteopath in Belgium is a male, between 40 and 49 years old, self-employed, owner of a clinic, with a part-time osteopathic training of 5 years, a prior education in physiotherapy and with a master’s degree in osteopathy or another healthcare profession.

### 4.1. Socio-Demographics

Although the osteopathic profession is predominantly male, which is in line with the 2010 [4] and 2013 [5] Belgian surveys, the number of female osteopaths is slightly increasing, with a gender shift in the 30–39 age group, which in 2013 was only seen in the 20–29 age group. The male dominance in the profession is also observed in other countries such as Italy (66.7%), Portugal (64%) and Spain (60%) [11,12,14], but contrasts with Austria, Germany and Switzerland, where there is a female predominance of 71%, 56.7% and 54.7%, respectively [7,8,13]. The median age of respondents in the three Belgian surveys is between 40–49 years, which is in line with Germany, the UK and Switzerland [7,8,17], but contrasts with the younger median age of 30–39 seen in Italy, Portugal and Spain [11,12,14].

According to all three Belgian surveys, most osteopaths practise in Flanders, a third work in Wallonia and 13% in Brussels. These figures correspond quite well with the population figures [18]. More generally, it can be said that the respondents are fairly well distributed throughout the country, with only a relative underrepresentation of respondents in the provinces of Limburg and Flemish Brabant and an overrepresentation in Walloon Brabant.

### 4.2. Osteopathic Education

Belgian osteopathic professional associations adhere to the European CEN Standard on training, and since 2014, only osteopaths with a master’s degree are eligible to join a professional association. Since the profession of osteopathy in Belgium is not yet regulated and education is a regional matter, there is only one French-speaking public university that currently, since 2004, offers a type I training in osteopathy (for those with little or no prior healthcare training). Their students follow a six-year university programme and graduate with a ‘master after master’ degree. Although the Chamber of Osteopathy, in preparation for the regularisation of the profession, with representatives of the various Dutch-speaking (Flemish) and French-speaking faculties of medicine, voted unanimously in favour of an osteopathic training programme within the universities [19], the Flemish universities refuse to open themselves up to non-conventional medicine [20]. There are four private osteopathic education institutes (OEI) in Belgium. At the time of this survey, only one of these OEI’s offered a five-year type I training in collaboration with a UK university, concluding with a master’s degree. All four provided type II training in osteopathy (aimed at those with prior training as healthcare professionals). Despite the claim of private institutions to be organised in accordance with the requirements of the CEN standard, a first analysis shows that even three years after the publication of the standard, a minimum of 1000 h of supervised osteopathic clinical practice is still a challenge [21]. This seems to be supported by the fact that 28% of respondents indicated that working in an osteopathic clinical setting was not part of their osteopathic training.

The number of respondents who indicated that they had received full-time osteopathic training rose from 23.7% in 2013 [5] to 34.9% in 2018. Whereas in 2010 [4] and 2013 [5], 83% of respondents had prior training in physical therapy, in 2018 this figure was 65.8%. Additionally, in Austria, Germany, Italy, Portugal and Spain, the majority of respondents received a part-time osteopathic training [8,9,11,12,13], and in Austria, Germany and Spain, the majority of respondents had prior training as a physical therapist [8,12,13]. Of all European countries surveyed [8,9,11,12,13], only in Belgium a majority of the respondents had a master’s degree; be it in osteopathy or another health care profession. The differences in master’s degrees between the Dutch- and French-speaking respondents in favour of the latter can be partially explained by the existence of a university programme in the French-speaking part of the country, whereby even two master’s degrees are obtained.

### 4.3. Osteopathic Identity and Practice Characteristics

As in several European countries [8,9,11,12,13,14] most participants showed a strong osteopathic identity, but only a majority of Italian (57%) and Belgian (81.6%) respondents advertised themselves exclusively as an osteopath. This clearly higher percentage in Belgium compared to other European countries can perhaps be explained by the fact that three out of four osteopathic professional associations do not allow their members to combine health professions. Respondents who were not members of a professional association were only 2.4% of 17.3% (Intermutualistic Agency non-members figure) and thus strongly underrepresented in this survey. In 2010 (*n* = 454) [4], 15.3% of all respondents combined osteopathy and physiotherapy, in 2013 (*n* = 702) [5] it was 25.8% and in the current survey, only 9.3% did. The difference between the percentages of 2010 and 2013 can be explained by the inclusion of a large number of osteopaths (*n* = 405) who were not members of a professional association and of a double number of respondents (*n* = 146) who were members of the only professional association that allows cumulation of these professions (UKO). The marked decrease in respondents who combine both professions in the current study corresponds well with the number of respondents who were not members of a professional association and those who were members of UKO. Moreover, the increase in the number of respondents who followed full-time training is certainly also a determining factor in this decrease. However, there were twice as many French-speaking respondents as Dutch-speaking respondents who combined both professions, whereas in 2013 the proportion was almost the same.

Consistent with previous studies, respondents of this study mostly worked alone as self-employed [4,5] and had consultations likely to last 30–45 min both for new and returning patients [5]. The median number of consultations per week of respondents of 31–35 is similar to the median number of 32 in 2013 [5]. The striking difference in the median number of consultations per week between the Dutch- and French-speaking respondents was also seen in the 2013 survey with a median of 30 consultations for French-speaking respondents and 35 for Dutch-speaking respondents. Compared to other countries surveyed, only Swiss respondents saw around 36 patients per week [7]. All others showed lower numbers with 28 in Germany, 26–30 in Spain, 21–25 in Austria and Portugal, and 11–15 in Italy [8,11,12,13,14].

### 4.4. Patient Profile

Despite the fact that the present study was practitioner-based, data regarding patients who visited an osteopath closely resembled the profile of patient-based studies [6,7,10,22,23]. In these patient-based studies, all ages were represented but the majority of patients who visited an osteopath were women between the ages of 40 and 65, with neck or lower back complaints. This profile corresponds with the data from previous osteopathic surveys [4,5] and with data from the 2018 Belgian National Health Survey [3]. The latter also showed that osteopaths were consulted significantly more by higher educated and residents of the French-speaking community. A survey of health and economic outcomes among individuals who received osteopathic care in 2018 (*n* = 2158) also found non-specific low back pain and non-specific neck pain as the two main complaints with which respondents consulted osteopaths at 32.4% and 17.7%, respectively. However, non-musculoskeletal complaints such as headaches and gastrointestinal complaints were also indicated as a main complaint for osteopathic consultation by respondents in 6.3% and 2.9%, respectively. The data were collected from a sample of members of the Socialist Health Insurance Fund, one of seven health insurance funds and the second largest in Belgium with about three million members (i.e., 30% of the population). Health insurance in Belgium is mandatory and residents can join one of those seven health insurance funds. Individuals aged 18–75 years with at least one osteopathic consultation in the first half of 2018 were included in this study [24].

### 4.5. Diagnostic and Therapeutic Modalities and Scope of Practice

Both in comparison with the Belgian 2013 survey [5] and with the other European surveys [7,8,11,12,13,14] it is clear that palpation of structures and of movement are the most commonly used diagnostic techniques in osteopathic practice, often complemented by palpation of tenderness or visual inspection. The five most commonly used osteopathic therapeutic techniques compared to those used in previous Belgian surveys [4,5] seem to be quite consistent. Only cranial techniques came in sixth and were replaced in the top five by myofascial techniques. Compared to other European surveys [7,8,11,12,13,14], only two therapeutic techniques featured most prominently in the top seven: articulatory [7,11,12,13,14] and visceral [7,8,13,14] techniques. The choice of visceral techniques is very different from that in the UK and Australia [10,17,23]. Thirty-two per cent of UK osteopaths declared to never use visceral techniques [17] and a recent study showed that only 5.1% applied these techniques during the first consultation [10].

An almost equal percentage of respondents as in the 2013 survey [5] used ‘supplementary methods’ in their osteopathic practice. So, it seems that a majority of Belgian osteopaths did not use supplementary methods in their practice, which was also the case for the Swiss respondents [7]. In both countries exercise therapy was the most used supplementary method for those who did use them. In contrast, the majority of respondents of the Spanish, Austrian, Portuguese and German surveys [8,11,12,13] used supplementary methods with 55.7%, 60.4%, 61.5% and 69.4%, respectively. In the UK, stretching exercise therapy is even applied in 32.9% and strengthening exercise therapy in 17% of a first consultation [10]. The same study shows that during a first consultation, patients are more likely to obtain dry needling (6.8%) than the application of a manual visceral technique (5.1%). All this means that the scope of the osteopathic practice in Europe is not always clear. This has consequences for the regulation of the profession in the different European countries, the curriculum of the training, the requirements for continuing professional development and the expectations of the patient towards the profession. Although the profession is not yet regulated in Belgium, the Chamber of Osteopathy, with equal representatives of the profession and of the faculties of medicine, has already worked out many proposals in this respect. Regarding their advice for the osteopathic scope of practice, ‘rehabilitation’ is on the list of unauthorised acts for osteopaths and thus reserved for physical therapists [19]. The fact that 41.5% of the 42.8% who use supplementary methods, still offer exercise therapy is mainly due to the fact that the majority of the respondents have had prior training as physiotherapists and that there are still 9.3% of respondents who combine both professions.

### 4.6. Cultural Differences

All but one of the survey sections showed considerable differences in the data between French and Dutch-speaking respondents. Only for ‘professional identity and views as an osteopath’ were the data very similar. Some of these differences may be explained by differences in osteopathic training and a greater familiarity with and trust in osteopathy in the French-speaking part of the country [25]. Other differences, such as the number of consultations per week, may be explained more by socio-economic and/or cultural differences.

### 4.7. Strength and Limitations

Although Belgian osteopaths have been surveyed before, this updated survey not only provides additional information regarding the professional identity of participants and their perceptions of the profession, but also modifies specific questions to improve its validity. However, limitations must be taken into account when discussing the findings of this study. Although the osteopathic profession has changed significantly in terms of its representation over the past decade, with some mergers leaving “only” four of the seven professional associations, the sample size may be skewed by the lack of a register of osteopaths required to provide data. Although we made every effort to also reach osteopaths who were not members of a professional association, only eight of the 255 contacts made available to us by the InterMutualistic Agency, responded to the questionnaire. Moreover, in 2013, the InterMutualistic Agency provided us with 405 contacts of non-member osteopaths. The chance that this number was reduced to 255 in 2018 is rather small. From this, it can be concluded that there are more osteopaths active in Belgium than the 1473 who were contacted by us for participation in the survey. Due to missing data and changed email addresses, these osteopaths could not be contacted.

Additionally, because osteopaths oversaw data entry, respondent bias may have affected the results. Although respondents were encouraged to refer to their diary/appointment calendar in the event of ambiguity, they were describing their practice, and it is unclear to what extent this information was based on audited clinical data.

## 5. Conclusions

This survey provided an update of the current profile of Belgian osteopaths, their socio-demographics, work situation and professional pursuits, training and continuing professional development, consultation fee and characteristics of clinical practice, profile of the patient, osteopathic skills and additional new information on their professional identity and views on the profession. The choice for the OPERA questionnaire for this survey facilitated international comparison. Compared to previous surveys, the feminisation of the profession seems to be slightly progressing with a gender shift to an older age group. Additionally, the number of osteopaths undergoing full-time training appears to have increased, while the number of osteopaths with prior training in physical therapy has decreased, leading to fewer combinations of the two professions. Despite the similarity in professional identity between the two linguistic regions in Belgium, there are quite a few differences concerning data from all other sections of this survey. This confirms the regional differences from the 2013 Benelux survey and calls for caution regarding possible socio-cultural influences. Finally, the information provided could contribute to the body of evidence used by stakeholders and policymakers for a long-awaited regulation of the profession in Belgium.

## Figures and Tables

**Table 1:** Distribution by gender, culture and age (*n* = 332).

Variable	*n*	%
Gender		
Male	228	68.7
Female	104	31.3
Cultural		
Dutch-speaking	177	53.3
French-speaking	155	46.7
Age		
20–29	30	9.0
30–39	88	26.5
40–49	89	26.8
50–59	67	20.2
60–65	31	9.3
>65	27	8.1

**Table 2:** Distribution of work situations (*n* = 332).

	Variable	*n*	%
Employment type	Self-employed	330	99.4
	Employee	2	0.6
	Both	0	0.0
Self-employed type	Owner of a clinic	269	81.5
	Business partner of a clinic	45	13.6
	Associate	16	4.9
Type of working collaboration	Alone	174	52.4
	Only as part of a team	69	20.8
	Both alone and as part of a team	89	26.8

**Table 3:** Distribution of participants’ consultation features (*n* = 332).

Time for a New Patient			Time for a Returning Patient		
Time (minutes)	*n*	%	Time (minutes)	*n*	%
<30	11	3.3	<30	28	8.4
30–45	173	52.1	30–45	228	68.7
46–60	137	41.3	46–60	76	22.9
>60	11	3.3	>60	0	0.0
**Fee first visit**			**Fee following visit**		
Fee (EUR)	*n*	%	Fee (EUR)	*n*	%
<41	8	2.4	<41	12	3.6
41–50	139	41.9	41–50	156	47.0
51–60	142	42.8	51–60	132	39.8
61–70	31	9.3	61–70	22	6.6
71–80	7	2.1	71–80	7	2.1
>80	5	1.5	>80	3	0.9
**Number of clinical working days/week**			**Number of patient consultations/week**		
Days	*n*	%	Patients	*n*	%
1	10	3.0	≤15	46	13.9
2	10	3.0	16–25	53	16.0
3	36	10.8	26–35	76	22.9
4	85	25.6	36–45	56	16.9
5	176	53.0	46–55	50	15.1
6	15	4.5	>56	51	15.4
**Average waiting time for first consultation**					
Variable	*n*	%			
Same day	13	3.9			
Next working day	46	13.9			
Within 2–7 working days	188	56.6			
Within 8–14 working days	56	16.9			
Between 2–4 weeks	21	6.3			
>1 Month	8	2.4			

**Table 4:** The 10 most common specific conditions (in descending order of ‘often’ and ‘very often’ responses).

	Never	Seldom	Regularly	Often	Very Often
Non-specific neck pain	0.0	1.5	4.2	28.6	65.7
Lumbar radiculopathy	0.0	0.9	5.1	35.8	58.1
Non-specific low back pain	0.0	1.2	4.8	27.4	66.6
Headache/and migraines	0.3	0.9	6.9	54.5	37.4
Cervical radiculopathy	0.9	0.6	16.3	46.7	35.5
Unsettled or crying babies (colic)	10.5	7.2	23.8	31.3	27.1
Gastro-oesophageal reflux	3.3	16.0	34.3	31.9	14.5
Digestive disorders	1.5	16.0	38.0	31.0	13.6
Complaints during/ after pregnancy	1.5	11.1	42.5	31.6	13.3
Irritable bowel syndrome	5.1	22.9	34.9	26.5	10.5

Numbers in table are %.

**Table 5:** The most common diagnostic procedures (in descending order of ‘often’ and ‘always’ responses).

Diagnostic Procedure	Never	Seldom	Regularly	Often	Always	Unknown
Palpation of position/structures	0.9	1.2	3.9	22.3	71.4	0.3
Palpation of movement	0.6	1.5	5.7	21.7	70.2	0.3
Palpation of tenderness	0.9	3.0	9.6	27.7	58.7	0.0
Assessment of visceral mobility	1.5	6.3	11.8	27.4	51.8	1.2
Visual inspection	6.0	6.9	9.6	20.2	56.0	1.2
Assessment of the cranium (neuro- and viscerocranium)	4.5	9.9	11.1	34.3	39.2	0.9
Muscle function testing	2.1	7.2	20.8	34.3	34.9	0.6
Neurologic testing	1.2	4.5	24.7	38.6	30.4	0.6
Orthopaedic testing	2.7	10.2	19.9	28.6	36.1	2.4
Fascial testing	4.2	9.0	21.4	35.2	27.4	2.7
Diagnostic imaging	7.8	11.5	28.0	31.9	15.0	5.7
Percussion and auscultation	9.6	18.1	28.6	26.5	15.7	1.5
Neurolymphatic reflex tests	22.3	19.9	24.1	15.4	7.2	11.1
Otoscopy	23.2	32.2	24.7	9.6	3.6	6.6
Blood analysis	26.0	28.6	24.4	7.5	1.5	12.1
Urinalysis	44.9	21.4	9.9	1.2	0.6	22.0

Numbers in table are %.

**Table 6:** The most common therapeutic procedures (in descending order of ‘often’ and ‘very often’ responses).

Therapeutic Procedure	Never	Seldom	Regularly	Often	Very Often	Unknown
Articulatory/mobilisation techniques (GOT/TBA)	1.2	1.5	6.6	23.5	66.9	0.3
Visceral techniques	1.8	6.3	13.6	29.9	46.7	1.8
HVLA	3.9	7.8	12.1	29.5	44.9	1.8
Myofascial techniques	7.2	6.0	12.1	36.8	37.1	0.9
Soft and connective tissue techniques	4.5	11.5	11.5	31.0	39.5	2.1
Neurocranial and viscerocranial techniques	5.4	6.3	14.8	33.1	37.4	3.0
Functional techniques	4.8	6.9	19.0	30.4	37.1	1.8
Progressive Inhibition of Neuromuscular Structures (PINS)	6.0	12.1	16.0	35.5	26.5	3.9
Muscle Energy Techniques	6.0	15.4	22.3	25	25	6.3
Fluid techniques	7.8	15.4	26.8	28.3	14.5	7.2
Automatic shifting and fluid body approach	16.0	12.1	15.7	17.5	16.3	22.6

Numbers in table are %; GOT: General Osteopathic Treatment; TBA: Total Body Adjustment; HVLA: High Velocity Low Amplitude.

## Data Availability

The data presented in this study are available in the present paper and its supporting information files.

## Supplementary Materials

The following supporting information can be downloaded at: https://www.mdpi.com/article/10.3390/healthcare10112136/s1, Figure S1: Colleagues of respondents working as part of a team; Table S1: Geographical distribution by province and membership in a professional osteopathic association (*n* = 332); Table S2: Other professional activities; Table S3: Osteopathic training and life-long learning characteristics (*n*= 332); Table S4: Osteopath identity statements; Table S5: Views as an osteopath statement.
