# Supplementary material for: The Profile of Belgian Osteopaths: A Cross-Sectional Survey
Source: Healthcare (Basel). 2022 Oct 27;10(11):2136. doi: 10.3390/healthcare10112136 (PMC9690369; doi:10.3390/healthcare10112136)
Supplement: Supplementary file 1 [file healthcare-10-02136-s001.zip › Supporting files/Table S1.pdf]

Table S1: Geographical distribution by province and membership in a professional osteopathic association (n=332).

Variable	n	%
**Geographical distribution by province**		
Antwerpen	55	16.6
Brussels	43	13.0
Vlaams Brabant	20	6.0
Brabant Wallon	32	9.6
Hainaut	34	10.2
Limburg	15	4.5
Liège	27	8.1
Luxembourg	6	1.8
Namur	16	4.8
West-Vlaanderen	44	13.3
Oost-Vlaanderen	40	12.1
**Professional osteopathic association**		
Yes	324	97.6
No	8	2.4
**Association**		
BUO/UBO	28	8.5
BVBO/UPOB	248	74.9
UKO	26	7.9
UPMO	28	8.5
ALDO	1	0.3
