# Supplementary material for: The Profile of Belgian Osteopaths: A Cross-Sectional Survey
Source: Healthcare (Basel). 2022 Oct 27;10(11):2136. doi: 10.3390/healthcare10112136 (PMC9690369; doi:10.3390/healthcare10112136)
Supplement: Supplementary file 1 [file healthcare-10-02136-s001.zip › Supporting files/Table S2.pdf]

Table S2: Other professional activities.

Activity	n	%
clinical physician	2	1.3
clinical physiotherapist	31	20.8
other healthcare practitioner	8	5.4
lecturing osteopathic courses in learning	38	25.5
supervising osteopathic clinical trainees	17	11.4
supervising osteopathic research dissertations	19	12.8
researcher	8	5.4
work for a professional osteopathic regulatory body or association	4	2.7
other professional activities	22	14.8
