# Supplementary material for: The Profile of Belgian Osteopaths: A Cross-Sectional Survey
Source: Healthcare (Basel). 2022 Oct 27;10(11):2136. doi: 10.3390/healthcare10112136 (PMC9690369; doi:10.3390/healthcare10112136)
Supplement: Supplementary file 1 [file healthcare-10-02136-s001.zip › Supporting files/Table S3.pdf]

Table S3: Osteopathic training and life-long learning characteristics (n= 332).

Descriptor	Variable	n	%
**Type of training**	Part-time	216	65.1
	Full-time	116	34.9
**Duration of the training**	3 years	19	5.7
	4 years	58	17.5
	5 years	149	44.9
	6 years	83	25.0
	>6 years	23	6.9
**Type of osteopathic academic qualification**	Diploma Osteopathy (DO)	218	65.7
	Bachelor (Graduate)	51	15.4
	Master	56	16.9
	PhD	6	1.8
**Continuous Professional Development (CPD)**	Yes	295	88.9
	No	37	11.1
**Previous qualification(s)**	Chiropractor	0	0.0
	Massage therapist	3	0.8
	Physician	4	1.1
	Midwife	0	0.0
	Nurse	1	0.3
	Physiotherapist	239	65.8
	Sport scientist	20	5.5
	Other healthcare training	12	3.3
	Other non-healthcare training	11	3.0
	No prior training	73	20.1
