# Supplementary material for: The Profile of Belgian Osteopaths: A Cross-Sectional Survey
Source: Healthcare (Basel). 2022 Oct 27;10(11):2136. doi: 10.3390/healthcare10112136 (PMC9690369; doi:10.3390/healthcare10112136)
Supplement: Supplementary file 1 [file healthcare-10-02136-s001.zip › Supporting files/Table S4.pdf]

Table S4: Osteopath identity statements.

Statement	strongly disagree	disagree	neither agree or disagree	agree	strongly agree
I strongly define myself as an osteopath.	0.3 (1)	1.2 (4)	3.9 (13)	16.0 (53)	78.6 (261)
Being an osteopath is important to me.	0.3 (1)	0.6 (2)	4.8 (16)	19.9 (66)	74.4 (247)
I'm proud to be an osteopath.	0.3 (1)	0.6 (2)	5.1 (17)	18.4 (61)	75.6 (251)
I strongly define myself as a health care practitioner.	2.4 (8)	0.9 (3)	4.8 (16)	21.4 (71)	70.5 (234)
Being a healthcare practitioner is important to me.	2.1 (7)	1.2 (4)	3.6 (12)	24.7 (82)	68.4 (227)

Numbers in table are % (n)
