# Supplementary material for: The Profile of Belgian Osteopaths: A Cross-Sectional Survey
Source: Healthcare (Basel). 2022 Oct 27;10(11):2136. doi: 10.3390/healthcare10112136 (PMC9690369; doi:10.3390/healthcare10112136)
Supplement: Supplementary file 1 [file healthcare-10-02136-s001.zip › Supporting files/Table S5.pdf]

Table S5: Views as an osteopath statements.

Statement	strongly disagree	disagree	neither agree or disagree	agree	strongly agree
Medical professionals (physicians, physiotherapists, etc.) in Belgium see osteopathy as a distinct healthcare discipline.	1.2<br>(4)	12.1<br>(40)	19.3<br>(64)	42.5<br>(141)	25.0<br>(83)
Overall the quality of patient care provided by osteopaths in Belgium is good.	0.0<br>(0)	2.1<br>(7)	9.9<br>(33)	62.1<br>(206)	25.9<br>(86)
Osteopathy should be regulated by law as an independent profession in Belgium.	1.2<br>(4)	1.8<br>(6)	7.8<br>(26)	19.6<br>(65)	69.6<br>(231)
Regulation would have a positive effect on how osteopaths practice.	2.4<br>(8)	4.8<br>(16)	19.6<br>(65)	23.2<br>(77)	50.0<br>(166)

Patients should be better reimbursed for osteopathic care in Belgium.	0.3 (1)	2.1 (7)	9.6 (32)	23.2 (77)	64.8 (215)
Medical professionals (physicians, physiotherapists, etc.) in Belgium see osteopathy as a subgroup of reimbursable manual therapy.	6.9 (23)	17.8 (59)	34.3 (114)	29.2 (97)	11.8 (39)
Osteopaths in Belgium would like to have better cooperation with other healthcare professionals.	0.3 (1)	2.1 (7)	8.1 (27)	36.1 (120)	53.3 (177)
Osteopathy in Belgium should be regulated as first line medical practice.	1.2 (4)	1.2 (4)	8.7 (29)	18.7 (62)	70.2 (233)
Osteopathy in Belgium should be regulated as an allied health profession (e.g. physiotherapy or occupational therapy).	53.3 (177)	14.2 (47)	13.9 (46)	10.5 (35)	8.1 (27)

Numbers in table are % (n)
