# Supplementary figures and images for: The Profile of Belgian Osteopaths: A Cross-Sectional Survey
Source: Healthcare (Basel). 2022 Oct 27;10(11):2136. doi: 10.3390/healthcare10112136 (PMC9690369; doi:10.3390/healthcare10112136)

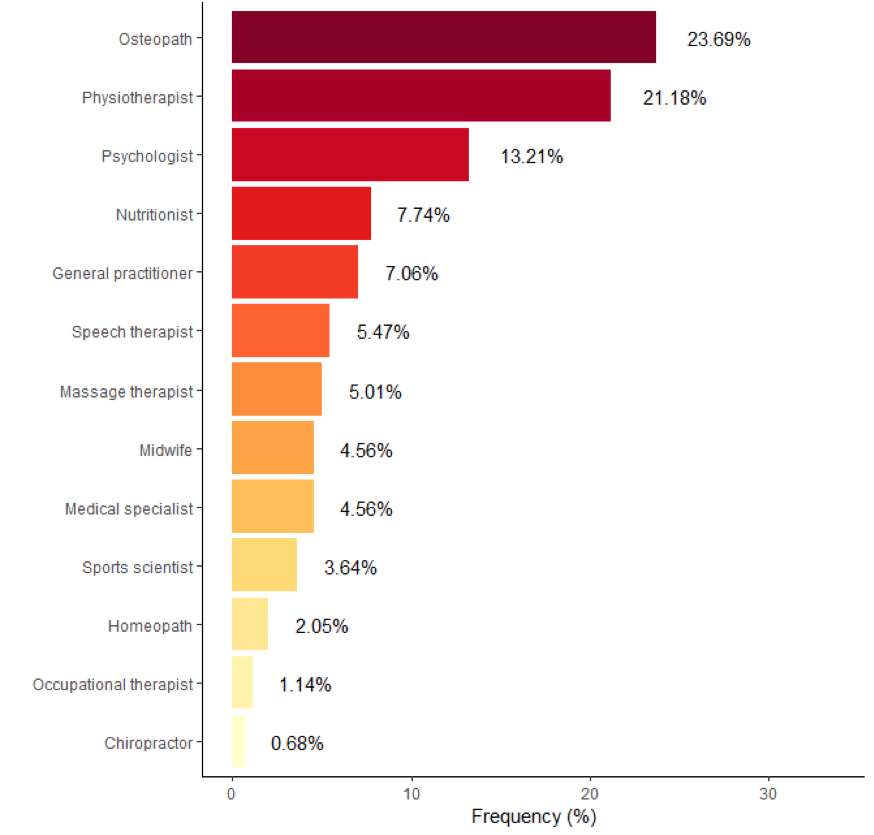

Supplement: Supplementary file 1 [file healthcare-10-02136-s001.zip › Supporting files/Figure S1.png]
